# Primary Care Physician Characteristics and Low-Value Care Provision in Japan

**DOI:** 10.1001/jamahealthforum.2025.1430

**Published:** 2025-06-06

**Authors:** Atsushi Miyawaki, John N. Mafi, Kazuhiro Abe, Alexandra Klomhaus, Rei Goto, Kei Kitajima, Daichi Sato, Yusuke Tsugawa

**Affiliations:** 1 Public Health Research Group, Institute of Medicine, University of Tsukuba, Tsukuba, Ibaraki, Japan; 2 Department of Health Services Research, Graduate School of Medicine, The University of Tokyo, Bunkyo-ku, Tokyo, Japan; 3 Department of Clinical Epidemiology and Health Economics, School of Public Health, The University of Tokyo, Bunkyo-ku, Tokyo, Japan; 4 Division of General Internal Medicine and Health Services Research, David Geffen School of Medicine, University of California, Los Angeles; 5 RAND Corporation, Santa Monica, California; 6 Division of Data-Based Health Management, Health Innovation and Technology Center, Faculty of Health Sciences, Hokkaido University, Sapporo, Hokkaido, Japan; 7 Graduate School of Business Administration, Keio University, Yokohama, Kanagawa, Japan; 8 M3 Inc, Akasaka Intercity, Minato-ku, Tokyo, Japan; 9 Department of Health Policy and Management, Fielding School of Public Health, University of California, Los Angeles

## Abstract

**Importance:**

Evidence is limited regarding the physician characteristics associated with the provision of low-value services in primary care, especially outside of the US.

**Objective:**

To measure physician-level use of 10 low-value care services that provide no net clinical benefit and to investigate the characteristics of primary care physicians who frequently provide low-value care in Japan.

**Design, Setting, and Participants:**

This cross-sectional analysis used a nationwide electronic health record database linked with claims data in Japan to assess visits by adult patients (age ≥18 years) to a solo-practice primary care physician from October 1, 2022, through September 30, 2023. Data analysis was performed from June 2024 to February 2025.

**Main Outcomes and Measures:**

Multivariable-adjusted composite rate of low-value care services delivered per 100 patients per year, aggregated across 10 low-value measures, after accounting for case mix and other characteristics.

**Results:**

Among 2 542 630 patients (mean [SD] age, 51.6 [19.8] years; 58.2% female) treated by 1019 primary care physicians (mean [SD] age 56.4 [10.2] years; 90.4% male), 436 317 low-value care services were identified (17.2 cases per 100 patients overall). Nearly half of these low-value care services were provided by 10% of physicians. After accounting for patient case mix, older physicians (age ≥60 years) delivered 2.1 per 100 patients (95% CI, 1.0-3.3) more low-value care services than those younger than 40 years; not board-certified physicians delivered 0.8 per 100 patients (95% CI, 0.2-1.5) more than general internal medicine board-certified physicians; physicians with higher patient volumes delivered 2.3 per 100 patients (95% CI, 1.5-3.2) more than those with low patient volumes; and physicians practicing in Western Japan delivered 1.0 per 100 patients (95% CI, 0.5-1.5) more than those in Eastern Japan.

**Conclusions and Relevance:**

The findings of this cross-sectional analysis suggest that low-value care services were common and concentrated among a small number of primary care physicians in Japan, with older physicians and not board-certified physicians being more likely to provide low-value care. Policy interventions targeting at a small number of certain types of physicians providing large quantities of low-value care may be more effective and efficient than those targeting all physicians uniformly.

## Key Points

**Question:**

How does low-value care (LVC) provision vary among primary care physicians and are there physician characteristics associated with the variability?

**Findings:**

This cross-sectional analysis including 2 542 630 patients in the electronic health record database used by Japanese primary care clinics found that 10 LVC services were provided 17.2 times per 100 adult patients annually. Nearly half of all LVC services were provided by 10% of physicians who were more likely to be older, not board certified, have high patient volumes, and practice in the Western regions.

**Meaning:**

These findings suggest that interventions targeted at the small number of certain types of physicians who have high LVC provision may be more effective for reducing LVC use and more efficient than targeting all physicians uniformly.

## Teaser

This cross-sectional analysis measures physician-level use of 10 low-value care services and investigates the characteristics of primary care physicians who frequently provide low-value care in Japan.

## Introduction

Low-value care (LVC)—health care services that provide no or little net clinical benefit to a patient in a specific clinical situation^1,2,3^—is increasingly recognized as a pervasive problem in health care systems worldwide.^4^ Decreased use of LVC has the potential to avoid unnecessary health spending, to improve quality of care by reducing overdiagnoses and overtreatment, and to reallocate spending to higher-value health care services that improve population health.^5^ Primary care physicians are uniquely positioned to play an important role in deimplementing LVC through their comprehensive understanding of patients’ values and preferences, ongoing relationships with patients, and responsibility for coordinating care.^6^

Various initiatives have aimed to raise general awareness about the issue of LVC.^7,8^ However, these interventions have often failed to achieve the desired results.^9,10,11,12^ Research indicates that most physicians do not provide LVC frequently enough to justify widespread interventions.^13,14,15,16^ Instead of a general educational campaign aimed at all physicians, a more individualized, physician-focused approach that includes a combination of audit and feedback, nudges, electronic clinical decision support, and education may be able to change physicians’ behavior toward reducing the provision of LVC.^17,18^

Interventions that focus on physicians who are more prone to providing LVC may be more effective in reducing LVC. However, there is limited evidence regarding the characteristics of physicians associated with providing LVC services in primary care.^15,19,20^ Existing studies on the topic focus on a narrow set of LVC services^15^ or are limited to older adults,^19,20^ resulting in an inadequate level of understanding of the factors affecting a broad set of LVC services across both older and younger adult populations.

Similar to the US, in Japan, LVC is an important health policy and public health issue, particularly as the country faces the dual challenge of ensuring fiscal sustainability and maintaining patient safety amid a rapidly aging population. Given that numerous LVC services are covered by public insurance plans^21,22^ and considering the limited knowledge of the physician-level factors that contribute to providing LVC,^6^ Japan may be experiencing higher health care spending and increased patient risk of adverse effects from LVC (eg, radiation exposure from redundant imaging studies, inappropriate procedures, and unnecessary health care expenditures). Moreover, Japan’s payment system, which combines government-regulated fee schedules (similar to the Medicare fee schedule in the US) and fee-for-service payment for outpatient care, may be serving as a structural driver of LVC.

Given that physicians play an important role in determining the type and quantity of health care services that patients receive, a better understanding of the physician characteristics associated with LVC provision should be informative for policymakers to develop interventions—such as training programs and payment reform—that have the potential to reduce wasteful health care spending.^17,18,23,24^ To address this critical knowledge gap, we examined the characteristics of physicians associated with LVC provision using a nationwide primary care clinic electronic health record (EHR) database linked with claims data in Japan.

## Methods

The University of Tokyo Ethics Committee approved this study and granted a waiver of written informed consent because we used deidentified data. We followed the Strengthening the Reporting of Observational Studies in Epidemiology (STROBE) reporting guideline the STROBE reporting guidelines for observational studies.^25^

### Study Context

Japan’s health system is characterized by predominantly private clinics and hospitals financed by a combination of social health insurance and cost-sharing from patients. Japanese citizens are required by law to enroll in 1 of the social health insurance plans, and regardless of the plan, beneficiaries are covered under standardized benefits, such as the same co-insurance rates (10%-30%, varies by age category), out-of-pocket maximum (covered by the catastrophic health insurance program), and the option to choose any hospital or clinic—similar to the Preferred Provider Organization plans in the US, except that in Japan, some tertiary hospitals charge an additional first-time visit fee for patients without a referral letter from their primary care physician. Insurance benefits are also standardized and include all health care services except for preventive services (which are financed using general tax revenues) and long-term care (covered under the long-term care insurance). More than 95% of clinics are privately operated, and most outpatient services are reimbursed through the fee-for-service model. Most inpatient care in large acute care hospitals is paid through a bundled payment called the Diagnosis Procedure Combination, which is a per-diem payment system based on diagnosis and procedures (a modified version of the diagnosis-related group in the US).

### Data Collection

We conducted a cross-sectional analysis using nationwide data from the Japan Medical Data Survey (JAMDAS), collected and compiled by M3 Inc (Tokyo, Japan).^26^ JAMDAS integrates EHR data with claims data from clinics across Japan, comprising all types of outpatient visits. From October 2022 to September 2023, JAMDAS recorded approximately 40 million visits at 3066 clinics across 46 of Japan’s 47 prefectures, representing approximately 4% of all primary care visits across the country.^27^ Compared to the national data from the official surveys in Japan,^28,29^ the characteristics of clinics included in JAMDAS were similar, except that physicians of the clinics included in the JAMDAS database were slightly younger and more frequently female (eTable 1 in Supplement 1). Although the sex distribution of patients registered in JAMDAS aligns closely with national estimates from the Patient Survey (a government statistical survey),^30^ those registered in JAMDAS tend to be slightly younger (eFigure 1 in Supplement 1).

The JAMDAS database includes data on primary care clinic, patient, and encounter variables. Clinic information includes the clinic director’s sex, age, and board certification, the number of physicians who work in the clinic, and the region where it is located.^16,31^ The database also includes clinic identifiers and information about the clinic in which the individual physicians work. For solo practice clinics, the database enabled us to identify individual physicians providing health care services, but for group practices, it did not specify which physician provided which health care services within a given clinic. It should be noted that any preventive services and screening tests (including both high-value and low-value services) were not recorded in our EHR data because, in Japan, these costs were covered by municipalities using the general tax revenues, rather than public health insurance.

### Study Population

We included all adult patients (age ≥18 years) who made at least 1 visit to any of the clinics continuously included in the JAMDAS database from October 1, 2022, through September 30, 2023 (hereafter, study year). In JAMDAS, patients can be tracked within the same clinic but not across different clinics; therefore, this database includes patient-clinic−level data as opposed to patient-level data. To focus on clinics treating patients who could potentially receive LVC services related to primary care, we restricted our analyses to clinics that treated at least 1 patient eligible to be included in the denominator for each measure of LVC service use assessed in this study. This process led to the exclusion of 39.1% of patients. To accurately attribute each patient to individual physicians, we restricted our analyses to patients who were treated by a solo-practicing primary care physician. This process led to the exclusion of 49.0% of the remaining patients (eFigure 2 in Supplement 1).

### LVC Service Measurement

We assessed each physician’s rates of provision of 10 LVC services that are typically provided in the primary care setting and reported these as the number of LVC services per 100 patients eligible for a measure’s denominator (hereafter, eligible patient) per year. We selected LVC services based on previously developed LVC service measures in Japan, established through consensus among physicians in 26 specialties.^21^ Focusing on primary care, we restricted our measure set to 7 services of the 33 services in the established set. These measures were based on the peer-reviewed medical literature and the Choosing Wisely campaign initiatives in Canada^32^ and the US .^7^ Furthermore, to assess the rate of LVC provision using a comprehensive set of LVC services in Japan, we conducted a predetermined literature review procedure. Two primary care physicians on the study team (A.M., K.A.) separately conducted literature reviews and reached consensus, identifying 3 additional LVC services commonly provided in primary care: mucolytic expectorants for acute upper respiratory infections (AURIs),^33^ codeine for AURIs,^34,35^ and vitamin B_12_ medications for diabetic neuropathy.^36^ The details of this process are described in eMethods 1 in Supplement 1. Ultimately, 10 LVC services were assessed, comprising 5 medications, 3 diagnostic or imaging tests, and 2 procedures.

Table 1 shows each LVC service measure, its operational definition, and the eligible denominator patient population.^33,34,35,36,37,38,39,40,41,42,43,44,45,46,47,48^ As previous studies have done,^19,49^ we used the more specific definition when both a sensitive (broader) and a specific (narrower) definition were available for defining a metric. This approach reduces the likelihood of detecting LVC services but also reduces the likelihood of misclassifying high-value services as LVC services. We used the definitions developed through the consensus method in previous research conducted in Japan,^21^ with some modifications for application to the JAMDAS data (eTable 2 in Supplement 1). For the 3 newly added LVC services (ie, mucolytic expectorants for AURIs, codeine for AURIs, and vitamin B_12_ medications for diabetic neuropathy), the definitions and measurements were established through a consensus method by 2 physicians (clinician-scientists) experienced in measuring health care services using Japanese claims data (A.M., K.A.), similar to the approach used in previous research.^21^

**Table 1.** Measures of Low-Value Care Services in Primary Care

Low-value care measure	Source	Operational definition	Denominator population (during study year)
Medication			
Expectorant for AURI	Literature review^33^	Acetylcysteine or carbocysteine prescription during an AURI visit without a new diagnosis for which antibiotics may be appropriate, or a coexisting diagnosis of chronic respiratory disease^a^^,^^b^	Patients with ≥1 AURI visit
Antibiotic for AURI	Literature review^37^	Oral antibiotics during AURI visit without a new diagnosis for which antibiotics may be appropriate^a^^,^^b^	Patients with ≥1 AURI visit
Codeine for AURI	Literature review^34,35^	Codeine prescription during an AURI visit without a new diagnosis for which antibiotics may be appropriate, or a coexisting diagnosis of chronic respiratory disease or chronic pain^a^^,^^b^	Patients with ≥1 AURI visit
Pregabalin for low back pain	Literature review^38,39^	Pregabalin prescription for patient with a diagnosis of back pain and without diagnosis of fibromyalgia, diabetes, postherpetic neuralgia, arteriosclerosis, disc disorder, trigeminal neuralgia, or peripheral neuropathy	Patients with a diagnosis of low back pain
Vitamin B_12_ medications for diabetic neuropathy	Literature review^36^	Vitamin B_12_ prescription for patient with diabetic neuropathy and without codiagnosis of vitamin B_12_ deficiency-related conditions	Patients with a diagnosis of diabetes
Laboratory or imaging tests			
Short-term repeat BMD testing	Literature review^40,41,42^	Second or subsequent BMD test for patient with a diagnosis of osteoporosis at the time of first BMD test in the year	Patients with a diagnosis of osteoporosis
Serum T3 level testing for hypothyroidism	CW^7^	Total or free T3 measurement for patient with a diagnosis of hypothyroidism	Patients with a hypothyroidism diagnosis
Unnecessary vitamin D testing	CW^7^	Vitamin D testing for patient without diagnosis of chronic kidney disease, disorders of calcium metabolism, secondary hyperparathyroidism, or vitamin-D deficiency and without diagnosis suggestive of non-PTH mediated hypercalcemia (sarcoidosis, tuberculosis, selected neoplasms)	All patients aged ≥18 y
Procedures			
Injection for low back pain	Literature review^43,44^	Epidural (not indwelling), facet, or trigger-point injection for patient with a diagnosis of low back pain and without diagnosis indicating radiculopathy	Patients with a diagnosis of low back pain
Unnecessary endoscopy for dyspepsia or constipation	CW Canada^32^ and literature review^45,46,47,48^	Endoscopy in patient aged 18-54 y with a diagnosis of dyspepsia and without a codiagnosis of dysphagia, anemia, weight loss, or digestive system cancer Colonoscopy in patient aged 18-49 with a diagnosis of constipation and without diagnosis of anemia, weight loss, digestive system cancer, or other digestive system disease	Patients aged 18-54 y with a diagnosis of dyspepsia or constipation

Abbreviations: AURI, acute upper respiratory infection; BMD, bone mineral density; CW, Choosing Wisely campaign; PTH, parathyroid hormone.

^a^ AURI visit was defined as a visit with a diagnosis of AURI and an index date corresponding to the date of the visit.

^b^ Diagnoses for which antibiotics may be appropriate: acute sinusitis, acute pharyngitis, tonsillitis, acute tracheitis, acute epiglottitis, acute bacterial pneumonia, unspecified acute lower-respiratory infection, peritonsillar abscess, chronic pharyngitis, chronic sinusitis, and otitis media.

### Primary Care Physician Characteristics

The physician characteristics included in the analyses were sex, age group (<40, 40-49, 50-59, ≥60 years), board-certified specialties, patient volume, and region (Eastern Japan [Hokkaido, Tohoku, and Kanto], Central Japan [Chubu and Kansai], and Western Japan [Chugoku, Shikoku, and Kyusyu/Okinawa]). The board-certified specialties were categorized into 3 groups: general internal medicine (termed *generalist*), specialties (51 specialties, termed *specialist*; further described in eMethods 2 in Supplement 1), and not board-certified physicians. For patient volume, we captured the median number of patients seen per practice-day per physician during a 1-year period, and then we used that distribution to calculate physician-level terciles for all the physicians included in the analyses. Based on regional differences in medical resources (eg, number of physicians and hospital beds) and medical spending per capita,^50^ we hypothesized that there could be regional variations in LVC provision.

### Statistical Analysis

First, we described patient and physician characteristics using mean (SD) for continuous variables and number (%) for categorical variables. Second, for each of the 10 indicators of LVC, we aggregated the number of services in the study year and calculated the prevalence (per 100 eligible patients and for patients overall). Third, to evaluate physician-level variation in LVC provision, we aggregated the numbers of LVC services for each physician and assessed their cumulative distributions. Fourth, we calculated an adjusted composite rate of LVC services delivered per 100 patients per year. To do this, we first used the sample of eligible patients for the 10 LVC measures and estimated the adjusted rates of LVC provision for each physician by separately running a multilevel Poisson regression analysis. We regressed the number of LVC services provided in the study year on the patient characteristics (sex, age [including linear, quadratic, and cubic terms], and Charlson Comorbidity Index score [0, 1, or ≥2]^51^) with physician random effects.^52^ We calculated Pearson correlation coefficients among these 10 LVC measures in individual physicians. Then, we calculated each physician’s adjusted composite rate as the weighted sum of the adjusted rate for the 10 measures, with weights based on the proportion of eligible patients in the entire patient population.^19^ This allowed interpretation of the composite rate as the expected number of LVC services per year that the physician would provide to 100 patients in a standardized primary care patient population (further described in eMethods 3 in Supplement 1). Lastly, we performed a physician-level multivariable linear regression analysis that regressed the composite rate of LVC services on the physician characteristics described previously. Standard errors were clustered at the prefecture level. Statistical tests were 2-tailed and *P* < .05 was considered as statistically significant. Data analyses were performed from June 2024 to February 2025 using Stata, release 17.0 (StataCorp).

### Sensitivity Analyses

We performed several sensitivity analyses. First, we tested the sensitivity to the model specification by using a generalized linear model with a log-link function.^53^ Second, we assessed the impact of high-volume LVC measures by dropping each of the 2 most common LVC measures. Third, we evaluated the influence of low-volume LVC measures by excluding the lower half of LVC measures in the absolute numbers from the composite rate calculation. Fourth, we changed the reference group to the largest physician category. Fifth, we weighted the analysis using the inverse of the estimated probability of each clinic’s inclusion in the JAMDAS database. This inclusion probability was calculated using clinic-level logistic regression, with inclusion in JAMDAS as the outcome, and clinic characteristics as variables in the sample of all Japanese medical institutions. The detailed calculation method for inclusion probability is described in the previous literature.^16^ Sixth, under Japan’s fee-for-service payment system, physicians are financially incentivized to perform more tests and procedures but not to prescribe more drugs. Therefore, to minimize the influence of financial incentives on LVC provision, we recalculated the composite rate using measures included in the drug category. Lastly, we tested generalizability by analyzing clinics overall—both solo practice clinics and group practice clinics—attributing the clinical practices in a group-practice clinic to the clinic director.

## Results

### Characteristics of Patients and Physicians

Our analysis included 1019 primary care physicians providing care to a total of 2 542 630 adult patients (mean [SD] age, 51.6 [19.8] years; 58% female and 42% male individuals) from October 2022 through September 2023 (Table 2). Of the physicians (mean [SD) age of 56.4 [10.2] years), 921 were male (90.4%) and 98 were female (9.6%).

**Table 2.** Characteristics of Patients and Physicians in Low-Value Care Study

Characteristic	No. (%)
Total patients, No.	2 542 630
Sex	
Female	1 478 907 (58.2)
Male	1 063 723 (41.8)
Age, mean (SD), y	51.6 (19.8)
Age category, y	
18-39	810 107 (31.9)
40-64	998 987 (39.3)
65-79	483 973 (19.0)
≥80	249 563 (9.8)
Charlson Comorbidity Index score	
0	1 895 681 (74.6)
1	342 173 (13.5)
≥2	304 776 (12.0)
Total physicians, No.	1019
Sex	
Female	98 (9.6)
Male	921 (90.4)
Age, mean (SD), y	56.4 (10.2)
Age category, y	
<40	45 (4.4)
40-49	247 (24.2)
50-59	309 (30.3)
≥60	418 (41.0)
Board-certified specialties^a^	
General internal medicine	99 (9.7)
Other specialties	459 (45.0)
Not board-certified	461 (45.2)
Patient volume^b^	
Low	338 (33.2)
Medium	356 (34.9)
High	325 (31.9)
Region in Japan	
Eastern	382 (37.5)
Central	437 (42.9)
Western	200 (19.6)

^a^ Detailed definitions of the board-certified specialties are described in eMethods 2 in Supplement 1.

^b^ Patient volume was defined as the terciles of the annual median number of patients and categorized as low (≤30 visits/d), medium (31-51 visits/d), or high (≥52 visits/d).

### LVC Provision

During the study year, we found that 436 317 LVC services were provided to 2 542 630 patients (17.2 times per 100 patients overall), and 10.9% of patients overall (276 622 of 2 542 630) received at least 1 LVC service. The 5 most frequent LVC services accounted for 95.7% of total LVC services: expectorants for AURIs (6.9 times per 100 patients overall), prescriptions of antibiotics for AURIs (5.0 times), injections for low back pain (2.0 times), codeines for AURIs (1.9 times), and pregabalin for low back pain (0.6 times) (Table 3).

**Table 3.** Breakdown of Low-Value Care (LVC) Episodes Among

LVC type	Annual LVC, No.	Eligible patients, No.	Annual LVC, patients, %		Patients receiving ≥1 LVC during the study year, No. (%)
			Eligible	Overall	
Medication					
Expectorant for AURI	174 880	552 792	31.6	6.9	159 153 (28.8)
Antibiotic for AURI	126 769	552 792	22.9	5.0	110 911 (20.1)
Codeine for AURI	49 446	552 792	8.9	1.9	46 454 (8.4)
Pregabalin for low back pain	15 369	201 635	7.6	0.6	2994 (1.5)
Vitamin B_12_ medications for diabetic neuropathy	3897	327 081	1.2	0.2	562 (0.2)
Laboratory or imaging tests					
Short-term repeat BMD testing	11 331	77 825	14.6	0.4	9649 (12.4)
Serum T3 level testing for hypothyroidism	2148	70 412	3.1	0.1	1156 (1.6)
Unnecessary vitamin D testing	48	2 542 630	0.002	0.002	48 (<0.01)
Procedures					
Injection for low back pain	51 103	201 635	25.3	2.0	10 209 (5.1)
Unnecessary endoscopy for dyspepsia or constipation	1326	62 904	2.1	0.1	1319 (2.1)

Abbreviations: AURI, acute upper respiratory infection; BMD, bone mineral density.

The denominator was the number of total patients (N = 2 542 630).

### Variation in LVC Provision Rates by Physician

LVC provision was skewed: 10% of primary care physicians who provided the most LVC services accounted for 45.2% of all LVC services (Figure). Examining correlations between pairs of adjusted LVC measures among physicians, we found significant positive correlations between 14 of 45 pairs (*r* = 0.07-0.24) (eTable 3 in Supplement 1). The median (IQR) of the adjusted composite rate of LVC provision across physicians was 13.9 (11.7-15.1) times per 100 patients overall per year (eFigure 3 and eTable 4 in Supplement 1).

**Figure.**
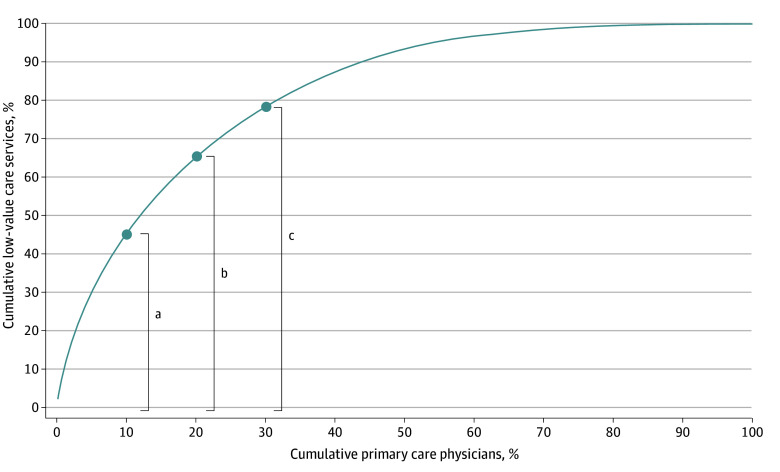
Distribution of Low-Value Care (LVC) Services Among Primary Care Physicians ^a^Top 10% of physicians accounted for 45.2% of all LVC provisions. ^b^Top 20% of physicians accounted for 65.5% of all LVC provisions. ^c^Top 30% of physicians accounted for 78.6% of all LVC provisions. We aggregated the number of LVC services for each primary care physician and assessed their cumulative distributions.

### Physician Characteristics and Composite Rate of LVC Provision

After accounting for patient case mix (Table 4), physicians 60 years or older delivered 2.1 LVC services per 100 patients per year (95% CI, 1.0-3.3) more than those younger than 40 years. Furthermore, not board-certified physicians delivered 0.8 LVC services (95% CI, 0.2-1.5) more than general internal medicine board-certified physicians, physicians with higher patient volumes delivered 2.3 (95% CI, 1.5-3.2) more than those with low patient volumes, and physicians in the Western region of Japan delivered 1.0 (95% CI, 0.5-1.5) more than those in the Eastern region. There was no evidence that the composite rate of LVC provision varied by male vs female sex. Our findings were qualitatively unaffected across various sensitivity analyses (data are available in eTables 5-11 in Supplement 1).

**Table 4.** Association of Physician Characteristics With the Composite Rate of Low-Value Care (LVC) Provision

Physician characteristic	No. of LVC services per 100 patients, adjusted difference (95% CI)^a^	*P* value
Sex		
Female	–0.4 (–1.1 to 0.4)	.34
Male	0 [Reference]	
Age, y		
<40	0 [Reference]	
40-49	2.0 (0.9 to 3.1)	.001
50-59	2.3 (1.1 to 3.4)	<.001
≥60	2.1 (1.0 to 3.3)	.001
Board-certified specialties		
General internal medicine	0 [Reference]	
Other specialties	–0.6 (–1.5 to 0.3)	.21
Not board certified	0.8 (0.2 to 1.5)	.01
Patient volume		
Low	0 [Reference]	
Medium	1.4 (0.8 to 2.1)	<.001
High	2.3 (1.5 to 3.2)	<.001
Region in Japan		
Eastern	0 [Reference]	
Central	0.5 (–0.1 to 1.1)	.13
Western	1.0 (0.5 to 1.5)	<.001

^a^ The multivariable linear regression model regressed the composite rate of LVC provision on physician characteristics (sex, age category, board-certified specialties, patient volume, and region where the physician practiced) and reported the coefficient for each variable.

## Discussion

Using EHR data linked with the claims data from primary care clinics across Japan, we found that LVC provision was concentrated among a small number of physicians. We also found that older physicians, not board-certified physicians, physicians with high patient volumes, and physicians practicing in the Western region of Japan were more likely to provide LVC. Taken together, our findings suggest that the policy interventions targeted at a small number of certain types of physicians providing a large quantity of LVC may be more effective and efficient compared with the interventions that target all physicians uniformly. A better understanding of the underlying mechanisms determining why certain types of physicians deliver more LVC should be informative for policymakers to develop more effective policy interventions that could modify physicians’ practice patterns. Furthermore, our findings indicate that personalized interventions may be both possible and more effective by tailoring approaches based on physicians’ characteristics.

Our study found that a large number of LVC services were provided in Japanese primary care (approximately 1 in 10 patients received at least 1 LVC service per year). This may be explained by several structural and institutional characteristics of Japan’s health care system, including a fee-for-service system for outpatient care, largely privately operated clinics and hospitals, and patients’ freedom to choose their clinics (free access).^24^ Although the Japanese government has the power to control the prices of health care services by changing the fee schedule, it has limited power to control the quantity of services provided. This has led to the overutilization of outpatient health care services, including a high utilization of outpatient-based LVC. Furthermore, the prevalence of LVC in the current study was higher than that found in our previous study, which measured 33 LVC services in a Japanese hospital setting (1 in 20 patients received at least 1 LVC service per year),^21^ possibly reflecting that some inpatient care is paid through a bundled payment rather than a fee-for-service payment.

We found that the top 5 LVC services accounted for more than 95% of the total volume. Among the 5 low-volume services, 4 (excluding endoscopies) were low-cost drugs or tests. While low-cost services may seem insignificant individually, research has shown that they can drive substantial unnecessary health care spending when provided at high volumes.^54^ Given the difference in overall budget impact, policy interventions focusing on frequently provided low-cost LVC may be more effective than those targeting infrequently provided high-cost LVC.

There are several mechanisms through which the provision of LVC differed by physician age and board certification status. First, not board-certified physicians and those for whom more time had elapsed since training may be practicing with outdated knowledge on medical overuse; they may have difficulty staying up to date with current guidelines.^55,56^ Second, the large difference found at age 40 years may be explained by the cohort effect among physicians; for example, given that evidence-based medicine was introduced in Japan around 2000,^57^ physicians trained before that period had limited exposure to this concept. While these physicians could learn evidence-based medicine by themselves, it may be difficult for some to keep up with the latest evidence without formal training. Relatedly, evidence-based clinical guidelines began development in Japan around the early 2000s,^57^ which may explain the difference in levels of LVC provision between older and younger physicians. Japan also introduced a formal postgraduate training program in 2004; before this, medical school graduates could become attending physicians or begin specialist training immediately after graduation. Given that medical students in Japan have limited clinical exposure during medical school, there were concerns about the preparedness of these newly graduated physicians. To address these concerns, in 2004 Japan implemented a mandatory postgraduate training program focusing on primary care,^58^ and the absence of this formal training among older physicians may explain their higher LVC use.

Our study found that physicians with higher daily patient volumes provided more LVC services per patient. Time constraints and/or mental exhaustion may affect physicians managing high patient volumes and prompt them to rely on low-value tests or treatments instead of conducting thorough medical history-taking and physical examinations. These challenges would be amenable to interventions to alleviate physicians’ time constraints and/or mental exhaustion while supporting or enhancing reserve capacity in physicians’ decision-making—eg, introduction of clinical decision support systems and further promotion of team-based practice. Alternatively, Japan’s fee-for-service payment system may incentivize more profit-oriented physicians, both to see a higher volume of patients and to provide LVC services. Finally, this association could be explained by a patient preference for physicians who provide more LVC because patients are concentrated among these physicians. However, given prior research suggesting that providing more LVC was not associated with higher patient experience ratings,^59^ this explanation would minimally explain this finding.

We found that more LVC was provided in the Western region of Japan, and this could be explained by a greater number of practicing physicians per capita in this region, potentially incentivizing the primary care physicians to overutilize health care services to attract more patients given the substantial competition.^60^ Other factors could include differences in patients’ health literacy levels^58^ and socioeconomic status,^61^ sales activities by pharmaceutical and medical device companies,^19,58^ and limited professional development opportunities.^62^ Future research should focus on exploring more localized regional differences while accounting for these factors.

Limited research has investigated physician characteristics and the provision of a broad set of LVC services. Bouck et al^15^ analyzed the provision of 4 low-value screening tests in administrative claims data in Ontario, Canada, finding that physicians who provided more LVC services were more likely to be male and relatively experienced. Schwartz et al^19^ reported that among US Medicare patients, the utilization rate of 17 LVC services was higher among male, older, or high-volume physicians. Barreto et al found that male, older, family medicine (vs internal medicine)−certified physicians as well as census region were associated with the higher spending of 8 LVC services. Our results exhibit a similar pattern, except regarding physician sex (likely due to the small proportion of female physicians), thus enhancing the generalizability of prior research. Despite fundamental differences in health insurance systems, medical malpractice litigation rates (potentially leading to defensive medicine), and health care culture and norms between Japan and the US, the highly concentrated distribution of LVC among a small number of physicians is similar in both countries. This finding suggests that LVC overuse may not be determined by the aforementioned country-specific factors, but rather by elements common to both health systems, such as the predominantly fee-for-service payment system that incentivizes health care practitioners to overutilize health care services, including LVC.^20^

### Limitations

Our study has limitations. First, as with any observational study, we could not eliminate the possibility of unmeasured confounding. Second, as with many studies directly measuring LVC,^1,63,64,65^ our analysis was limited by our use of administrative health care data. While the JAMDAS database accurately captures service provision, it lacks the detailed clinical information necessary to assess the appropriateness of these services. To mitigate this uncertainty, we prioritized specificity in identifying LVC, minimizing the likelihood of care being erroneously categorized as low value. Despite this limitation, the measurement of LVC through administrative data offers a more cost-effective approach for continuous monitoring than manual reviews of clinician records. Third, our findings could have potentially limited generalizability to primary care physicians not included in JAMDAS. While this was a convenience sample, the demographic characteristics of patients largely reflected national estimates for Japan. Lastly, our findings were limited to primary care in Japan, and therefore, may not be generalizable to other contexts, including hospital inpatient and emergency department care and health systems in other countries.

## Conclusions

This cross-sectional analysis of 10 LVC services in Japanese primary care settings found that LVC provision was concentrated among a small number of physicians, highlighting the potential for targeted policy interventions. We also found that physicians who were older, not board-certified, seeing a high volume of patients, and/or practicing in the Western region of Japan were more likely to provide LVC. A better understanding of the underlying mechanisms driving these patterns in high-risk physician groups could facilitate the development and dissemination of optimal interventions to reduce LVC.

## Notes

Supplement 1. eMethod 1. Process to Identify Additional Low-Value Care (LVC) services eMethod 2. Categorization of Board-Certified Specialties eMethod 3. Calculation of an Adjusted Composite Rate of Low-Value Care Services eFigure 1. Comparison of the Distributions of Patient Sex and Age for Primary Care Visits Between the Patient Survey and the JAMDAS eFigure 2. Sample Selection Flowchart eFigure 3. Physician-Level Variation in Low-Value Care Provision eTable 1. Comparison of the Clinic Characteristics Between the JAMDAS and the Nationwide Data eTable 2. Codes for Measures of Low-Value Care Services eTable 3. Correlations Between Low-Value Care Measures eTable 4. Distribution of Adjusted Rate of Provision of Low-Value Care for Each of the 10 Measures eTable 5. Association Between Physician Characteristics and Composite Rate of Low-Value Care Provision, Using A Generalized Linear Model Instead of Using A Linear Regression Model eTable 6. Association Between Physician Characteristics and Composite Rate of Low-Value Care Provision, Excluding the High-Volume Low-Value Care from the Composite Rate Calculation eTable 7. Association Between Physician Characteristics and the Composite Rate of Low-Value Care Provision, Excluding the Lower Half of Measures in the Absolute Numbers from the Composite Rate Calculation eTable 8. Association Between Physician Characteristics and the Composite Rate of Low-Value Care Provision, Changing the Reference Group to the Category with the Largest Physician Sample Size eTable 9. Association Between Physician Characteristics and the Composite Rate of Low-Value Care Provision, Weighting the Analysis Using the Inverse of the Estimated Probability of Inclusion in JAMDAS (Inclusion Probability). eTable 10. Association Between Physician Characteristics and Composite Rate of Low-Value Care Provision, Using Only Measures Included in the Drug Category for the Low-Value Care Composite Rate Calculation eTable 11. Association Between Physician Characteristics and Composite Rate of Low-Value Care Provision, Among Clinics Overall (Including Both Solo Practice Clinics and Group Practice Clinics) eReference

Supplement 2. Data Sharing Statement
