# Supplementary material for: Primary Care Physician Characteristics and Low-Value Care Provision in Japan
Source: JAMA Health Forum. 2025 Jun 6;6(6):e251430. doi: 10.1001/jamahealthforum.2025.1430 (PMC12144622; doi:10.1001/jamahealthforum.2025.1430)
Supplement: Supplement 1. — eMethod 1. Process to Identify Additional Low-Value Care (LVC) services eMethod 2. Categorization of Board-Certified Specialties eMethod 3. Calculation of an Adjusted Composite Rate of Low-Value Care Services eFigure 1. Comparison of the Distributions of Patient Sex and Age for Primary Care Visits Between the Patient Survey and the JAMDAS eFigure 2. Sample Selection Flowchart eFigure 3. Physician-Level Variation in Low-Value Care Provision eTable 1. Comparison of the Clinic Characteristics Between the JAMDAS and the Nationwide Data eTable 2. Codes for Measures of Low-Value Care Services eTable 3. Correlations Between Low-Value Care Measures eTable 4. Distribution of Adjusted Rate of Provision of Low-Value Care for Each of the 10 Measures eTable 5. Association Between Physician Characteristics and Composite Rate of Low-Value Care Provision, Using A Generalized Linear Model Instead of Using A Linear Regression Model eTable 6. Association Between Physician Characteristics and Composite Rate of Low-Value Care Provision, Excluding the High-Volume Low-Value Care from the Composite Rate Calculation eTable 7. Association Between Physician Characteristics and the Composite Rate of Low-Value Care Provision, Excluding the Lower Half of Measures in the Absolute Numbers from the Composite Rate Calculation eTable 8. Association Between Physician Characteristics and the Composite Rate of Low-Value Care Provision, Changing the Reference Group to the Category with the Largest Physician Sample Size eTable 9. Association Between Physician Characteristics and the Composite Rate of Low-Value Care Provision, Weighting the Analysis Using the Inverse of the Estimated Probability of Inclusion in JAMDAS (Inclusion Probability). eTable 10. Association Between Physician Characteristics and Composite Rate of Low-Value Care Provision, Using Only Measures Included in the Drug Category for the Low-Value Care Composite Rate Calculation eTable 11. Association Between Physician Characteristics and Co [file jamahealthforum-e251430-s001.pdf]

Supplemental Online Content

Miyawaki A, Mafi JN, Abe K, et al. Primary Care Physician Characteristics and Low-Value Care Provision in Japan. *JAMA Health Forum.* Published online June 6, 2025. doi:10.1001/jamahealthforum.2025.1430

eMethod 1. Process to Identify Additional Low-Value Care (LVC) services

eMethod 2. Categorization of Board-Certified Specialties

eMethod 3. Calculation of an Adjusted Composite Rate of Low-Value Care Services

eFigure 1. Comparison of the Distributions of Patient Sex and Age for Primary Care Visits Between the Patient Survey and the JAMDAS

eFigure 2. Sample Selection Flowchart

eFigure 3. Physician-Level Variation in Low-Value Care Provision

eTable 1. Comparison of the Clinic Characteristics Between the JAMDAS and the Nationwide Data

eTable 2. Codes for Measures of Low-Value Care Services

eTable 3. Correlations Between Low-Value Care Measures

eTable 4. Distribution of Adjusted Rate of Provision of Low-Value Care for Each of the 10 Measures

eTable 5. Association Between Physician Characteristics and Composite Rate of Low-Value Care Provision, Using A Generalized Linear Model Instead of Using A Linear Regression Model

eTable 6. Association Between Physician Characteristics and Composite Rate of Low-Value Care Provision, Excluding the High-Volume Low-Value Care from the Composite Rate Calculation

eTable 7. Association Between Physician Characteristics and the Composite Rate of Low-Value Care Provision, Excluding the Lower Half of Measures in the Absolute Numbers from the Composite Rate Calculation

©2025 Miyawaki A et al. *JAMA Health Forum*. All rights reserved, including those for text and data mining, AI training, and similar technologies.

eTable 8. Association Between Physician Characteristics and the Composite Rate of Low-Value Care Provision, Changing the Reference Group to the Category with the Largest Physician Sample Size

eTable 9. Association Between Physician Characteristics and the Composite Rate of Low-Value Care Provision, Weighting the Analysis Using the Inverse of the Estimated Probability of Inclusion in JAMDAS (Inclusion Probability).

eTable 10. Association Between Physician Characteristics and Composite Rate of Low-Value Care Provision, Using Only Measures Included in the Drug Category for the Low-Value Care Composite Rate Calculation

eTable 11. Association Between Physician Characteristics and Composite Rate of Low-Value Care Provision, Among Clinics Overall (Including Both Solo Practice Clinics and Group Practice Clinics)

eReference

This supplemental material has been provided by the authors to give readers additional information about their work.

©2025 Miyawaki A et al. *JAMA Health Forum*. All rights reserved, including those for text and data mining, AI training, and similar technologies.

eMethod 1. Process to identify additional low-value care (LVC) services

In this process to identify additional LVC services, we followed a pre-determined literature review procedure. First, two primary care physicians on the study team (A.M. and K.A.) separately searched for and identified potential low-value care candidates in primary care widely provided in Japan (27 items) along with clinical evidence. These two physicians were also familiar with quantifying services in healthcare administrative data. Second, we excluded 2 duplicated measures. Third, for the remaining 25 items, A.M. and K.A. independently re-evaluated the clinical evidence for the LVC candidates proposed by each other and reached consensus, thereby selecting items that were definitely LVC (i.e. there is evidence that it has no clinical benefit, namely the service has been concluded to be "having no effect" in multiple randomized controlled trials or meta-analyses). This process excluded 20 items. In doing so, if A.M. and K.A. did not reach a consensus, a third physician (Y.T.) from the research team made the decision. Finally, among the remaining five items, those that could be plausibly measured in primary care adult sample in JAMDAS data were extracted (three items) by A.M., K.A., and Y.T. For the three newly added LVC services, definitions were established through a consensus method by two physicians (clinician-scientists) experienced in measuring healthcare services in Japanese claims data (A.M. and K.A.).

©2025 Miyawaki A et al. *JAMA Health Forum*. All rights reserved, including those for text and data mining, AI training, and similar technologies.

eMethod 2. Categorization of Board-Certified Specialties

As of 2020, Japan's Ministry of Health, Labour and Welfare recognized 56 medical specialty board certifications that could be advertised under the Medical Care Act. The JAMDAS maintains records of these specialty certifications for clinic owners, with some physicians holding multiple specialties. Our analytic data included data on 52 of these medical specialty board certifications (as shown in the column "inclusion criteria" in the table below).

We categorized physicians based on their board-certified specialties as follows: (1) the general internal medicine (GIM) group included physicians holding a GIM board certification, regardless of any additional certifications (GIM is the only board certification for generalists among the recorded medical specialty board certifications); (2) the "other specialties" group comprised physicians without a GIM board certification but holding another specialty; and (3) the "non-board-certified" group included physicians without any of the above certifications.

In the Japanese primary care system, many primary care physicians receive specialist training in hospitals before transitioning to primary care.[1] Furthermore, due to the scarcity of a well-developed training program in primary care or family medicine in Japan to date, physicians can open primary care practices regardless of their specialty and even without holding any board certification.[2] This has resulted in a low proportion of generalists in Japan, similar to South Korea and Taiwan, compared to other high-income countries.[3,4] According to the National Physician Census in 2022,[5] 7.3% of physicians of primary care clinics held a GIM board certification, 56.4% held a board certification in other specialties, and 36.3% were non-board-certified.

Category	Board-certified specialties	
	**Inclusion criteria**	**Exclusion criteria**
General internal medicine (GIM)	GIM	-
Other specialties	Allergic diseases; Cardiovascular disease; Diabetes; Endocrinology and metabolism; Gastroenterology; Geriatric medicine; Hematology; Hepatology; Infectious disease; Nephrology; Neurology; Pulmonary disease; Rheumatology; Anesthesiology; Breast care; Bronchoscopy; Burn care; Cardiovascular surgery; Cerebral endovascular therapy; Chinese medicine; Clinical genetics; Colorectal disease; Cytology; Dermatology; Dialysis; Emergency medicine; Gastroenterological surgery; Gastrointestinal endoscopy; General surgery; Gynecologic oncology; Laser therapy; Neurosurgery; Nuclear medicine; Obstetrics and gynecology; Oncology drug therapy; Ophthalmology; Orthopedics; Otorhinolaryngology; Pain medicine; Pathology; Pediatric neurological diseases; Pediatrics; Perinatal (neonatal) medicine; Physiatry (rehabilitation); Plastic surgery; Psychiatry; Psychiatric liaison; Radiology; Bronchoesophagology; Ultrasonography; Urology	GIM
Non-board-certified	Physicians without any of the above certifications	-

©2025 Miyawaki A et al. *JAMA Health Forum*. All rights reserved, including those for text and data mining, AI training, and similar technologies.

eMethod 3. Calculation of An Adjusted Composite Rate of Low-Value Care Services

In this study, we calculated the adjusted composite rate of LVC services by using patient-level analyses and then examined the association between physician characteristics and the composite rate by using a physician-level analysis.

To calculate the adjusted composite rate of LVC services delivered per 100 patients per year, we first estimated the adjusted rates of LVC provision by each physician for each of the 10 LVC measures separately through risk adjustment using indirect standardization.[6] For each LVC measure, we used a sample of eligible patients and conducted a patient-level multilevel Poisson regression analysis (e.g., for antibiotics for acute upper respiratory infections [AURIs], we use a sample of patients with at least 1 visit for AURIs). In this regression, we regressed the number of LVC services provided in the study year for each patient on patient characteristics (sex, age [including linear, quadratic, and cubic terms], and Charlson Comorbidity Index score [0, 1, or ≥ 2]). Poisson regression was used because the outcome variable was count data. Physician random effects were also included to deal with sampling error in physicians with a small patient size and non-random sorting of patients to physicians. After running each regression, we calculated each physician's "expected LVC provision rate" by averaging the values predicted by using the "predict" command for the physician.

Next, we calculated each physician's "observed LVC provision rate" by dividing the number of LVC services provided by the physician by the total number of patients treated by that physician. We then computed the Observed-to-Expected (O/E) ratio by dividing the observed LVC provision rate by the expected LVC provision rate. Finally, we determined the physician's adjusted rate of LVC provision by multiplying the O/E ratio by the number of LVC services delivered per 100 patients in the total eligible sample. This adjusted rate serves as a component score for the composite score calculation.

Since there were 10 LVC measures, we repeated this process 10 times, one for each measure. It is important to note that the analyzed patient samples varied across the LVC measures, as the eligible patients differed for each measure. When calculating physician-level composite scores by summing the physician-level component scores for each low-value service, we weighted each component score by the proportion of eligible patients in the entire patient population.[7] This weighting scheme allows the composite score to be interpreted as the number of LVC services that the physician would be expected to provide per year to 100 patients in a standardized primary care patient population. For example, the contribution of a component score to the composite score would be the same whether a service is used once per patient in the entire patient population or twice per patient in half of the population. Because the composite rate was a continuous variable, we used a linear regression model in the physician-level analysis that investigated the association between physician characteristics and the composite rate.

©2025 Miyawaki A et al. *JAMA Health Forum*. All rights reserved, including those for text and data mining, AI training, and similar technologies.

eFigure 1. Comparison of the Distributions of Patient Sex and Age for Primary Care Visits Between the Patient Survey and the JAMDAS

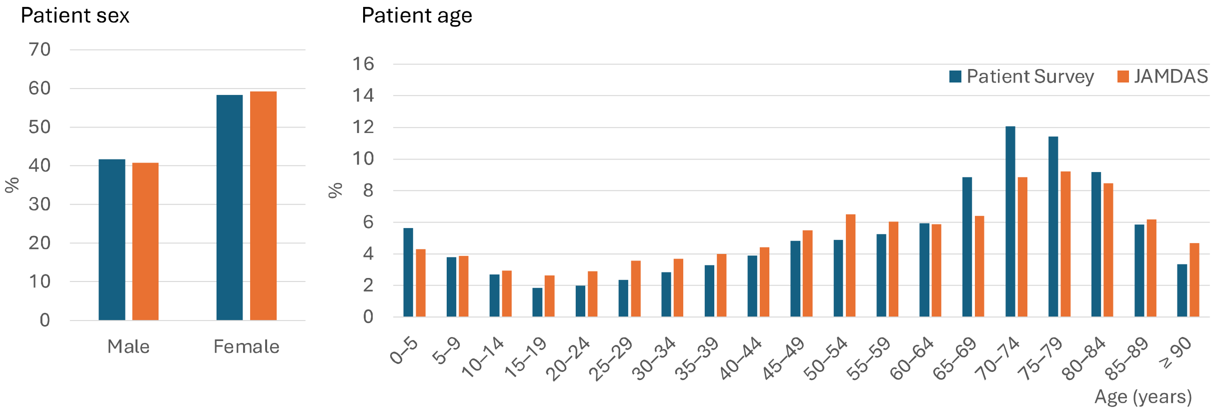

The unweighted patient sex and age distributions for all visits across the 3066 medical institutions continuously enrolled in the JAMDAS from October 2022 through September 2023 were compared against the nationwide estimates derived from the Patient Survey conducted in 2020 (estimates were based on clinics sampled through stratified random sampling across Japan). In comparison to the estimates for primary care visits from the Patient Survey, the patients registered in the JAMDAS exhibited a similar sex composition and tended to be slightly younger in age.

©2025 Miyawaki A et al. *JAMA Health Forum*. All rights reserved, including those for text and data mining, AI training, and similar technologies.

eFigure 2. Sample Selection Flowchart

8 192 554 patients aged ≥ 18 years in 2936 clinics

↓ We excluded 3 207 312 patients who visited the 1190 clinics that did not have a single patient who was eligible as a denominator for each of the indicators of low-value services in primary care.*

4 985 242 patients in 1746 primary care clinics

↓ We excluded 2 442 612 patients who visited 727 group-practice clinics.†

2 542 630 patients in 1019 solo practice primary care clinics

* Excluding patients treated by physicians with zero contribution to the LVC denominator resulted in an exclusion of only 20 generalists (from 176 to 156, a 11.4% reduction), indicating that most generalists contributed to denominators of all the low-value care indicators.

† The JAMDAS data could not distinguish between physicians within a clinic, although the characteristics of the clinic owner were available. Therefore, we were only able to accurately attribute practices to physician characteristics in solo practice clinics. However, in a sensitivity analysis, we repeated our analyses among clinics overall (including both solo practice clinics and group practice clinics). For group practice clinics, we attributed the clinical practices to the clinic owners, assuming that clinic owners' preferences and directives significantly influence the clinic's practices.

©2025 Miyawaki A et al. *JAMA Health Forum*. All rights reserved, including those for text and data mining, AI training, and similar technologies.

eFigure 3. Physician-Level Variation in Low-Value Care Provision

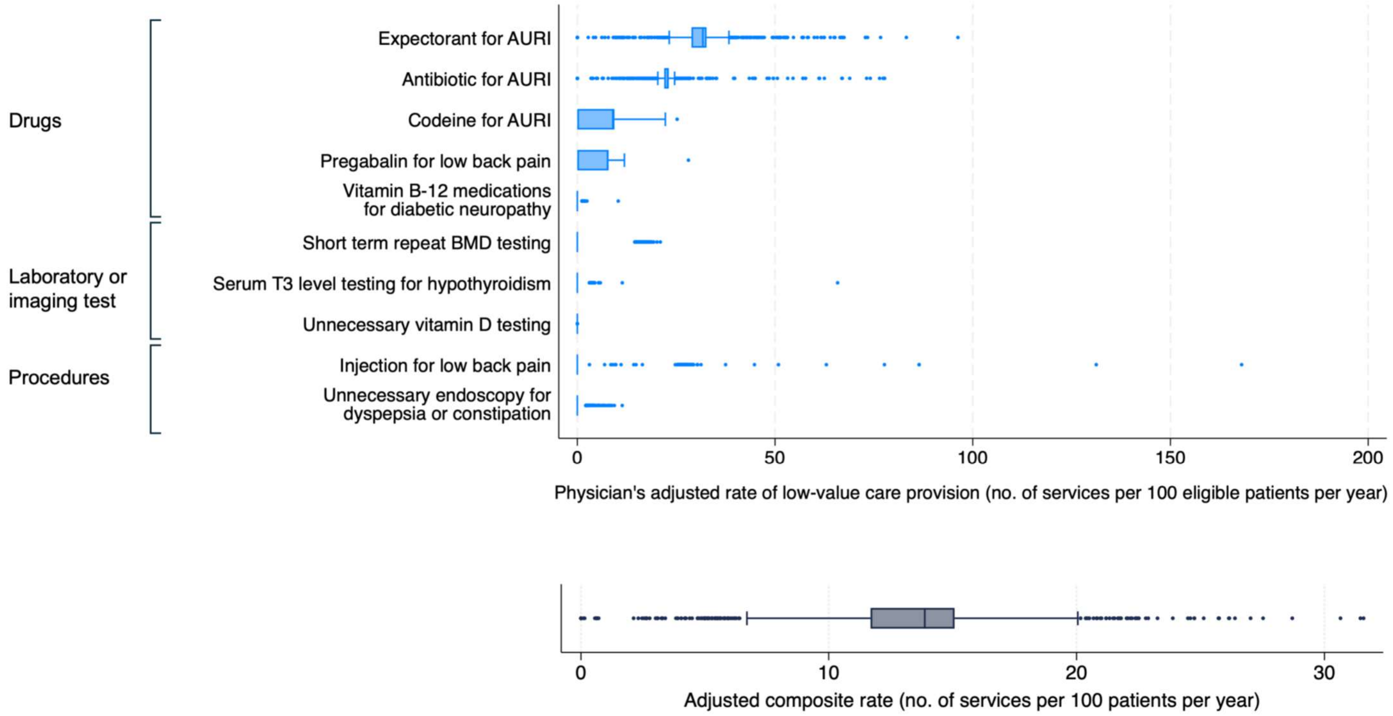

Abbreviations: AURI, Acute upper respiratory infection; BMD test, Bone mineral density test.
To estimate the adjusted rate of provision of low-value care (LVC) by each physician (i.e., adjusted number of LVC services provided in the study year per 100 eligible patients) for the 10 low-value measures, we applied a patient-level multilevel Poisson regression model that adjusted for the patient characteristics in the sample of eligible patients for each measure (the top panel). For each physician, we also calculated a composite rate of LVC provision by calculating the weighted sum of the adjusted rates across the 10 measures (the bottom panel). We weighted each component by the proportion of eligible patients in the entire adult patient population, allowing the interpretation of the composite rate as the total number of LVC services per year that the physician would be expected to provide to 100 persons in a standardized primary care patient population. The ends of the boxes represent the 25th (Q1) and 75th (Q3) percentile values. The middle line represents the median (50th percentile, Q2). The whiskers extend to the minimum and maximum values within 1.5 times the interquartile range (IQR) from Q1 and Q3. The dots represent outliers, which are values more than 1.5 times the IQR from Q1 or Q3.

©2025 Miyawaki A et al. *JAMA Health Forum*. All rights reserved, including those for text and data mining, AI training, and similar technologies.

eTable 1. Comparison of the Clinic Characteristics Between the JAMDAS and the Nationwide Data

	**JAMDAS data***	**National data†**
No. of working physicians, mean	1.7	1.5
Physicians' female sex, %	15.7	12.4
Physicians' age, mean, years, %	56.1	62.5
Generalist clinic, %	5.8	6.9
Region, %		
Eastern	43.5	43.1
Central	37.9	35.9
Western	18.6	21.0

*Estimates for physicians who own the clinics included in the JAMDAS data.
†National data are derived from Statistics of Physicians, Dentists and Pharmacists in 2022[5] and Survey of Medical Institutions.[8]

©2025 Miyawaki A et al. *JAMA Health Forum*. All rights reserved, including those for text and data mining, AI training, and similar technologies.

eTable 2. Codes for Measures of Low-Value Care Services

Low-value care	Codes for identification
Expectorant for acute upper respiratory infection (AURI)	**Anatomical Therapeutic Chemical classification system code (ATC code):** R05CB01(acetylcysteine), R05CB03 (carbocysteine)<br>**International Classification of Diseases 10th Revision (ICD-10) code**: J00–J06 (AURIs); J01 (acute sinusitis); J02.0 (streptococcal pharyngitis); J03.0 (streptococcal tonsillitis); J04.1 (acute tracheitis); J05.1 (acute epiglottitis); J13–J18 (bacterial pneumonia); J22 (unspecified acute lower respiratory infection); J31.2 (chronic pharyngitis); J32 (chronic sinusitis); J36 (peritonsillar abscess); H66 (otitis media); C33, C34, J41–J45, J47, J60–J69, J84 (chronic respiratory disease)
Antibiotic for AURI	**ATC code:** J01 (antibiotics)<br>**ICD-10 code:** J00–J06 (AURIs); J01 (acute sinusitis); J02.0 (streptococcal pharyngitis); J03.0 (streptococcal tonsillitis); J04.1 (acute tracheitis); J05.1 (acute epiglottitis); J13–J18 (bacterial pneumonia); J22 (unspecified acute lower respiratory infection); J31.2 (chronic pharyngitis); J32 (chronic sinusitis); J36 (peritonsillar abscess); H66 (otitis media)
Codeine for AURI	**ATC code:** N02AA08, N02AA58, R05DA04, R05DA12 (codeine)<br>**ICD-10 code:** J00–J06 (AURIs); J01 (acute sinusitis); J02.0 (streptococcal pharyngitis); J03.0 (streptococcal tonsillitis); J04.1 (acute tracheitis); J05.1 (acute epiglottitis); J13–J18 (bacterial pneumonia); J22 (unspecified acute lower respiratory infection); J31.2 (chronic pharyngitis); J32 (chronic sinusitis); J36 (peritonsillar abscess); H66 (otitis media); C33, C34, J41–J45, J47, J60–J69, J84 (chronic respiratory disease); R52.1, R52.2 (chronic pain)
Pregabalin for low back pain	**ATC code:** N02BF02 (pregabalin)<br>**ICD-10 code:** M43.0, M43.1, M51.0, M51.1, M51.2, M51.3, M51.8, M51.9, M53.3, M53.9, M54.3, M54.4, M54.5, M54.9, M96.1, M99, Q76.2, S33 (low back pain); M79.7 (fibromyalgia); B02.2, G53.0 (postherpetic neuralgia); M50, M51 (disc disorder); G50 (trigeminal neuralgia); E10-E14 (diabetes); I70 (arteriosclerosis); T81.2, G54–G64 (peripheral neuropathy)
Vitamin B-12 medications for diabetic neuropathy	**ATC code:** B03BA04, B03BA05 (vitamin B-12 medications)<br>**ICD-10 code:** E10–E14 (diabetes); D51, D52, E53.8, E53.9 (vitamin B-12 or folic acid deficiency); E10.4, E10.7, E11.4, E11.7, E13.4, E13.7, E14.4, G59.0, G63.2 (diabetic neuropathy)
Short term repeat bone mineral density testing	**Japanese Medical Practice Code:** 160091310, 160186870, 160147310, 160170410 (mineral density test)<br>**ICD-10 code:** M80, M81, M82 (osteoporosis)
Serum T3 level testing for hypothyroidism	**Japanese Medical Practice Code:** 160031310, 160033210 (serum T3 test)<br>**ICD-10 code:** E02, E03 (hypothyroidism)
Unnecessary vitamin D testing	**Japanese Medical Practice Code:** 160158150<br>**ICD-10 code:** N18.1, N18.2, N18.3 (chronic kidney disease); E83.5 (disorders of calcium metabolism); E21.1 (secondary hyperparathyroidism); E55 (vitamin D deficiency); D86 (sarcoidosis); J65, A15-A19, B90 (tuberculosis); C43, C44, C50, C56, C64, C65, C67, C81-C86, C88, C90-C96 (selected neoplasms)
Injection for low back pain	**Japanese Medical Practice Code:** 150235510, 150236010, 150242110, 150266010, 150265010, 150235710, 150350710, 150236010, 150265710, 150351310, 150239110<br>**ICD-10 code:** M43.0, M43.1, M51.0, M51.1, M51.2, M51.3, M51.8, M51.9, M53.3, M53.9, M54.3, M54.4, M54.5, M54.9, M96.1, M99, Q76.2, S33 (back pain); M47.2, M51.1, M54.1 (radiculopathy)
Unnecessary endoscopy for dyspepsia or constipation	**Japanese Medical Practice Code:** 160093810 (esophagogastroduodenoscopy), 160094710, 160094810, 160094910, 160202750 (colonoscopy)<br>**ICD-10 code:** F45.3, K30, R10.1 (dyspepsia) K58.9, K59.0 (constipation); D50-53, D55-64 (anemia), R13.1 (dysphagia), R63.4, R64 (weight loss); C15-26, C78.4-78.8 (cancer of digestive system), K20-K31, K35-K38, K40-K44, K50-K52, K55-K64 (except for K58.9 and K56.0), K65-K67, K70-K77, K80-K87, K90-K93 (other diseases of the digestive system)

The operational definitions were based on claims information from the electronic health record, including the *Anatomical Therapeutic Chemical* (ATC) *Classification* codes, the *International Classification of Diseases 10th Revision* (ICD-10) diagnosis codes, and the *Japanese Medical Practice Codes*, a collection of standardized codes representing medical procedures, supplies, products, and services used in Japan.[9] As was the case with the previous Japanese study conducted by our team,[9] definitions derived from prior studies in the United States[10] were applied for four LVC services (antibiotics for AURIs, short term repeat bone mineral density testing, serum T3 level testing for hypothyroidism, unnecessary vitamin D testing, and injection for low back pain). For unnecessary endoscopy for dyspepsia or constipation, a definition derived from prior studies in the Australia[11] was used. In doing so, measurement algorithms were adapted to better capture these services using the Japanese claims data. For pregabalin for low back pain, we applied definitions derived from our previous study.[9] For expectorants for AURIs, codeine for AURIs, and vitamin B-12 medications for diabetic neuropathy (three newly added LVC services), definitions were developed through a consensus method by two physician (clinician-scientists) experienced in measuring healthcare services using Japanese claims data, following the approach used in previous research.[9]

©2025 Miyawaki A et al. *JAMA Health Forum*. All rights reserved, including those for text and data mining, AI training, and similar technologies.

eTable 3. Correlations Between Low-Value Care Measures

	Expectorant for AURI	Antibiotic for AURI	Codeine for AURI	Pregabalin for low back pain	Vitamin B-12 medications for diabetic neuropathy	Short term repeat bone mineral density testing	Serum T3 level testing for hypothyroidism	Unnecessary vitamin D testing	Injection for low back pain	Unnecessary endoscopy for dyspepsia or constipation
Expectorants for acute upper respiratory infections (AURI)	1									
Antibiotics for AURI	0.168*	1								
Codeine for AURI	0.217*	0.208*	1							
Pregabalin for low back pain	0.068*	0.102*	0.097*	1						
Vitamin B-12 medications for diabetic neuropathy	0.082*	0.073*	0.122*	0.037	1					
Short term repeat bone mineral density testing	0.016	0.009	0.033	0.088*	0.058	1				
Serum T3 level testing for hypothyroidism	–0.002	–0.002	0.024	0.019	0.129*	–0.008	1			
Unnecessary vitamin D testing	0.039	–0.008	–0.056	0.016	–0.032	0.130*	0.040	1		
Injection for low back pain	0.012	–0.050	–0.019	0.239*	–0.018	0.212*	–0.025	–0.010	1	
Unnecessary endoscopy for dyspepsia or constipation	0.023	0.034	0.059	0.025	0.027	0.027	0.004	0.082*	–0.004	1

*, *P* value < 0.05. We calculated Pearson's correlation coefficient for each pair of 10 low-value care measures (adjusted rate of provision of low-value care) in individual physicians.

©2025 Miyawaki A et al. *JAMA Health Forum*. All rights reserved, including those for text and data mining, AI training, and similar technologies.

eTable 4. Distribution of Adjusted Rate of Provision of Low-Value Care For Each of the 10 Measures

	**Physician's adjusted rate of provision of low-value care (adjusted number of services per 100 eligible patients per year)**		
	Range	Mean (SD)	Median (IQR)
Expectorant for acute upper respiratory infection (AURI)	0–96.3	27.7 (14.4)	31.8 (28.9–32.7)
Antibiotic for AURI	0–77.7	21.7 (9.0)	22.9 (22.0–23.1)
Codeine for AURI	0–25.3	6.1 (5.0)	9.0 (0–9.4)
Pregabalin for low back pain	0–28.1	4.3 (4.1)	7.7 (0–7.9)
Vitamin B-12 medications for diabetic neuropathy	0–10.4	0.2 (0.6)	0 (0–0)
Short term repeat bone mineral density testing	0–21.0	3.5 (6.6)	0 (0–0)
Serum T3 level testing for hypothyroidism	0–65.9	0.3 (2.3)	0 (0–0)
Unnecessary vitamin D testing	0–0.01	0.4*10^4 (1.3)	0 (0–0)
Injection for low back pain	0–168.0	6.0 (13.3)	0 (0–0)
Unnecessary endoscopy for dyspepsia or constipation	0–11.4	0.4 (1.3)	0 (0–0)

Abbreviation: SD, standard deviation; IQR, interquartile range.

©2025 Miyawaki A et al. *JAMA Health Forum*. All rights reserved, including those for text and data mining, AI training, and similar technologies.

eTable 5. Association Between Physician Characteristics and Composite Rate of Low-Value Care Provision, Using A Generalized Linear Model Instead of Using A Linear Regression Model

Physician characteristics	Incidence rate ratio [a](95%CI)	*P* value
**Sex**		
Male	Reference	
Female	0.97 (0.92 to 1.03)	.34
**Age, years**		
< 40	Reference	
40–49	1.17 (1.07 to 1.28)	.001
50–59	1.19 (1.09 to 1.30)	< .001
≥ 60	1.18 (1.08 to 1.30)	.001
**Board-certified specialties**		
General internal medicine	Reference	
Other specialties	0.96 (0.89 to 1.03)	.21
Non-board-certified	1.07 (1.01 to 1.12)	.01
**Patient volume**		
Low	Reference	
Medium	1.12 (1.06 to 1.18)	< .001
High	1.20 (1.13 to 1.27)	< .001
**Region**		
Eastern	Reference	
Central	1.04 (0.99 to 1.09)	.11
Western	1.08 (1.04 to 1.12)	< .001

[a] We regressed the composite rate of low-value care provision on physician characteristics (sex, age category, board-certified specialties, patient volume, and region where the physician practiced). To account for non-negative distribution of the outcome, we used a multivariable generalized linear model with a log-link function (instead of a linear regression model), where we allowed the outcome variable to depend on its mean.[10] Standard errors were clustered at the prefecture level. This model, also known as a quasi-maximum likelihood estimation Poisson regression model, has been plausibly used for non-negative zero-inflated continuous outcome (e.g., medical expenditure).[11,12] We reported the incidence rate ratio (IRR) for each variable. IRR indicates the multiplicative change in the rate of provision of low-value care for the one-unit change of each exposure. Namely, IRR 1.2 indicates that the rate of provision of low-value care for a physician increase by 1.2 times on average.

©2025 Miyawaki A et al. *JAMA Health Forum*. All rights reserved, including those for text and data mining, AI training, and similar technologies.

eTable 6. Association Between Physician Characteristics and Composite Rate of Low-Value Care Provision, Excluding the High-Volume Low-Value Care from the Composite Rate Calculation

	(1) Excluding expectorants for AURIs		(2) Excluding antibiotics for AURIs	
**Physician characteristics**	**Adjusted difference (95% CI), [a] (n of services per 100 patients)**	***P* value**	**Adjusted difference (95% CI), [a] (n of services per 100 patients)**	***P* value**
**Sex**				
Male	Reference			
Female	–0.4 (–0.8 to +0.1)	.10	–0.3 (–1.0 to +0.4)	.37
**Age, years**				
< 40	Reference		Reference	
40–49	+1.3 (+0.3 to +2.2)	.01	+1.0 (–0.02 to +2.0)	.06
50–59	+1.4 (+0.7 to +2.1)	< .001	+1.2 (+0.1 to +2.4)	.04
≥ 60	+1.8 (+1.0 to +2.6)	< .001	+0.8 (–0.2 to +1.8)	.11
**Board-certified specialties**				
General internal medicine	Reference		Reference	
Other specialties	–0.3 (–0.8 to +0.2)	.19	–0.2 (–1.0 to +0.6)	.66
Non-board-certified	+0.7 (+0.2 to +1.2)	.01	+0.6 (+0.5 to +1.2)	.03
**Patient volume**				
Low	Reference		Reference	
Medium	+0.7 (+0.3 to +1.0)	< .001	+1.0 (+0.4 to +1.7)	.002
High	+1.3 (+0.8 to +1.9)	< .001	+1.8 (+1.2 to +2.3)	< .001
**Region**				
Eastern	Reference		Reference	
Central	+0.4 (+0.1 to +0.7)	.02	+0.3 (–0.1 to +0.8)	.15
Western	+0.6 (+0.3 to +1.0)	.001	+0.7 (+0.2 to +1.2)	.003

Abbreviation: AURIs, acute upper respiratory infections.

[a] We excluded (1) expectorants for AURIs or (2) antibiotics for AURIs when calculating composite rate of low-value care provision, and repeated analyses. We performed a multivariable linear regression model that regressed the composite rate on physician characteristics (sex, age category, board-certified specialties, patient volume, and region where the physician practiced) and reported the coefficient for each variable.

©2025 Miyawaki A et al. *JAMA Health Forum*. All rights reserved, including those for text and data mining, AI training, and similar technologies.

eTable 7. Association Between Physician Characteristics and the Composite Rate of Low-Value Care Provision, Excluding the Lower Half of Measures in the Absolute Numbers from the Composite Rate Calculation

Physician characteristics	Adjusted difference (95% CI), [a] (n of services per 100 patients)	*P* value
**Sex**		
Male	Reference	
Female	–0.3 (–1.1 to +0.5)	.44
**Age, years**		
< 40	Reference	
40–49	+1.9 (+0.8 to +3.1)	.001
50–59	+2.2 (+1.1 to +3.3)	< .001
≥ 60	+2.1 (+0.9 to +3.2)	.001
**Board-certified specialties**		
General internal medicine	Reference	
Other specialties	–0.6 (–1.5 to +0.3)	.22
Non-board-certified	+0.8 (+0.2 to +1.5)	.02
**Patient volume**		
Low	Reference	
Medium	+1.4 (+0.7 to +2.0)	< .001
High	+2.2 (+1.4 to +3.1)	< .001
**Region**		
Eastern	Reference	
Central	+0.5 (–0.2 to +1.1)	.16
Western	+1.0 (+0.4 to +1.5)	< .001

[a] From the composite score calculation, we excluded the Lower Half of Measures in the Absolute Numbers, including vitamin B-12 medications for peripheral nerve disorder due to diabetes, short term repeat bone mineral density testing, serum T3 level testing for hypothyroidism, unnecessary vitamin D testing, unnecessary endoscopy for dyspepsia or constipation, and repeated analyses. We performed a multivariable linear regression model that regressed the composite rate of low-value care provision on physician characteristics (sex, age category, board-certified specialties, patient volume, and region where the physician practiced) and reported the coefficient for each variable.

©2025 Miyawaki A et al. *JAMA Health Forum*. All rights reserved, including those for text and data mining, AI training, and similar technologies.

eTable 8. Association Between Physician Characteristics and the Composite Rate of Low-Value Care Provision, Changing the Reference Group to the Category with the Largest Physician Sample Size

Physician characteristics	Adjusted difference (95% CI), [a] (n of services per 100 patients)	*P* value
**Sex**		
Male	Reference	
Female	–0.4 (–1.1 to +0.4)	.34
**Age, years**		
< 40	–2.1 (–3.3 to –1.0)	.001
40–49	–0.1 (–0.8 to +0.5)	.65
50–59	+0.1 (–0.8 to +1.0)	.77
≥ 60	Reference	
**Board-certified specialties**		
General internal medicine	–0.8 (–1.5 to –0.2)	.01
Other specialties	–1.4 (–2.1 to –0.8)	< .001
Non-board-certified	Reference	
**Patient volume**		
Low	–1.4 (–2.1 to –0.8)	
Medium	Reference	< .001
High	+0.9 (+0.2 to +1.7)	.02
**Region**		
Eastern	–0.5 (–1.1 to +0.1)	.13
Central	Reference	
Western	+1.0 (–0.1 to +1.2)	.10

[a] We performed a multivariable linear regression model that regressed the composite rate of low-value care provision on physician characteristics (sex, age category, board-certified specialties, patient volume, and region where the physician practiced) and reported the coefficient for each variable.

©2025 Miyawaki A et al. *JAMA Health Forum*. All rights reserved, including those for text and data mining, AI training, and similar technologies.

eTable 9. Association Between Physician Characteristics and the Composite Rate of Low-Value Care Provision, Weighting the Analysis Using the Inverse of the Estimated Probability of Inclusion in JAMDAS (Inclusion Probability).

Physician characteristics	Adjusted difference (95% CI), [a] (n of services per 100 patients)	*P* value
**Sex**		
Male	Reference	
Female	–0.6 (–2.3 to +1.1)	.46
**Age, years**		
< 40	Reference	
40–49	+2.0 (+0.6 to +3.3)	.005
50–59	+2.1 (+0.8 to +3.5)	.003
≥ 60	+1.7 (+0.1 to +3.2)	.04
**Board-certified specialties**		
General internal medicine	Reference	
Other specialties	–0.8 (–1.8 to +0.3)	.14
Non-board-certified	+1.0 (0 to +2.1)	.05
**Patient volume**		
Low	Reference	
Medium	+1.5 (+0.7 to +2.4)	.001
High	+2.6 (+1.7 to +3.5)	< .001
**Region**		
Eastern	Reference	
Central	+0.9 (–0.1 to +2.0)	.08
Western	+1.6 (+0.6 to +2.7)	.003

[a] In this sensitivity analysis, we weighted the analysis using the inverse of the estimated probability of inclusion in JAMDAS (inclusion probability), which was calculated using clinic-level logistic regression with inclusion in JAMDAS as the outcome and clinic characteristics as variables in the sample of all Japanese medical institutions. The detailed calculation method for the inclusion probability is described in the previous literature (reference no. 16 in the main text).

©2025 Miyawaki A et al. *JAMA Health Forum*. All rights reserved, including those for text and data mining, AI training, and similar technologies.

eTable 10. Association Between Physician Characteristics and Composite Rate of Low-Value Care Provision, Using Only Measures Included in the Drug Category for the Low-Value Care Composite Rate Calculation

Physician characteristics	Adjusted difference (95% CI), [a] no. of services per 100 patients	*P* value
**Sex**		
Male	Reference	
Female	–0.1 (–0.9 to +0.6)	.71
**Age, years**		
< 40	Reference	
40–49	+1.9 (+0.8 to +3.0)	.001
50–59	+2.0 (+0.9 to +3.0)	< .001
≥ 60	+1.8 (+0.7 to +3.0)	.002
**Board-certified specialties**		
General internal medicine	Reference	
Other specialties	–0.9 (–1.8 to +0.1)	.07
Non-board-certified	+0.5 (–0.2 to +1.2)	.17
**Patient volume**		
Low	Reference	
Medium	+1.3 (+0.7 to +1.9)	.30
High	+1.9 (+1.1 to +2.7)	< .001
**Region**		
Eastern	Reference	
Central	+0.2 (–0.4 to +0.9)	.49
Western	+0.7 (+0.2 to +1.1)	< .001

To minimize the influence of financial incentives on provision of low-value care, this sensitivity analysis calculated the composite rate using only measures included in the drug category, and then we repeated the analyses. We performed a multivariable linear regression model that regressed the composite rate on physician characteristics (sex, age category, board-certified specialties, patient volume, and region where the physician practiced) and reported the coefficient for each variable.

©2025 Miyawaki A et al. *JAMA Health Forum*. All rights reserved, including those for text and data mining, AI training, and similar technologies.

eTable 11. Association Between Physician Characteristics and Composite Rate of Low-Value Care Provision, Among Clinics Overall (Including Both Solo Practice Clinics and Group Practice Clinics)

Physician characteristics	Adjusted difference (95% CI), [a] (n of services per 100 patients)	*P* value
**Sex**		
Male	Reference	
Female	–0.06 (–0.20 to +0.07)	.36
**Age, years**		
< 40	Reference	
40–49	+0.38 (+0.12 to +0.63)	.004
50–59	+0.41 (+0.14 to +0.68)	.004
≥ 60	+0.40 (+0.15 to +0.64)	.002
**Board-certified specialties**		
General internal medicine	Reference	
Other specialties	–0.12 (–0.30 to +0.05)	.17
Non-board-certified	+0.14 (–0.01 to +0.29)	.06
**Patient volume**		
Low	Reference	
Medium	+0.43 (+0.35 to +0.52)	< .001
High	+0.54 (+0.42 to +0.66)	< .001
**Region**		
Eastern	Reference	
Central	+0.01 (–0.09 to +0.11)	.87
Western	+0.13 (+0.05 to +0.22)	.003

[a] In this sensitivity analysis, we repeated our analyses among clinics overall (including both solo practice clinics and group practice clinics, rather than for only solo practice clinics). For group practice clinics, we attributed the clinical practices to the clinic owners, assuming that clinic owners' preferences and directives significantly influence the clinic's practices. We analyzed 1746 clinics. We performed a multivariable linear regression model that regressed the composite rate on clinic owner's characteristics (sex, age category, board-certified specialties), the clinic's patient volume (low [≤ 33 visits per day], middle [34–56 visits per day], or high [≥ 57 visits per day]), and region where the clinic was located and reported the coefficient for each variable.

©2025 Miyawaki A et al. *JAMA Health Forum*. All rights reserved, including those for text and data mining, AI training, and similar technologies.

eReference

1. Matsumoto M, Inoue K, Farmer J, Inada H, Kajii E. Geographic distribution of primary care physicians in Japan and Britain. *Health and Place*. 2010;16(1):164-166. doi:10.1016/j.healthplace.2009.07.005
2. Yamamoto Y, Haruta J, Goto R, Maeno T. What kinds of work do Japanese primary care physicians who derive greater positive meaning from work engage in? A cross-sectional study. *J Gen Fam Med*. 2023;24(2):94-101. doi:10.1002/jgf2.595
3. Cheng TM. International Health Care System Profiles: Taiwan. International Health Care System Profiles. June 5, 2020. Accessed September 30, 2024. https://www.commonwealthfund.org/international-health-policy-center/countries/taiwan
4. Kwon S. Case studies: Republic of Korea. In: *Price Setting and Price Regulation in Health Care: Lessons for Advancing Universal Health Coverage*. OECD Publishing; 2019. https://wkc.who.int/docs/librariesprovider24/wkc-projects-documents/2-6-case-study-rep-of-korea.pdf?sfvrsn=158712f4_1. Accessed February 23, 2025.
5. Ministry of Health Labour and Welfare Japan. Statistics of Physicians, Dentists and Pharmacists 2022. https://www.mhlw.go.jp/toukei/saikin/hw/ishi/22/index.html. Accessed February 23, 2025.
6. Center for Medicare and Medicaid Services. *Statistical Issues In Assessing Hospital Performance*.; 2012.
7. Schwartz AL, Jena AB, Zaslavsky AM, McWilliams JM. Analysis of physician variation in provision of low-value services. *JAMA Intern Med*. 2019;179(1):125. doi:10.1001/jamainternmed.2018.5086
8. Ministry of Health Labour and Welfare. Survey of Medical Institutions. 2023. Accessed February 23, 2025. https://www.mhlw.go.jp/toukei/list/79-1.html. Accessed February 23, 2025.
9. Services for Information of Medical Fee. Japanese Medical Practice Codes, Master File. https://shinryohoshu.mhlw.go.jp/shinryohoshu/searchMenu/doSearchInputSp. Accessed February 18, 2025.
10. Wooldridge JM. *Econometric Analysis of Cross Section and Panel Data*. MIT Press; 2010.
11. Chandra A, Gruber J, McKnight R. The impact of patient cost-sharing on low-income populations: Evidence from Massachusetts. *J Health Econ*. 2014;33(1):57-66. doi:10.1016/j.jhealeco.2013.10.008
12. Buntin MB, Zaslavsky AM. Too much ado about two-part models and transformation ? Comparing methods of modeling medicare expenditures. *J Health Econ*. 2004;23:525-542. doi:10.1016/j.jhealeco.2003.10.005

©2025 Miyawaki A et al. *JAMA Health Forum*. All rights reserved, including those for text and data mining, AI training, and similar technologies.
