# Supplementary material for: Primary Care Physician Characteristics and Low-Value Care Provision in Japan
Source: JAMA Health Forum. 2025 Jun 6;6(6):e251430. doi: 10.1001/jamahealthforum.2025.1430 (PMC12144622; doi:10.1001/jamahealthforum.2025.1430)
Supplement: Supplement 2. — Data Sharing Statement [file jamahealthforum-e251430-s002.pdf]

Data Sharing Statement

Miyawaki. Primary Care Physician Characteristics and Low-Value Care Provision in Japan. *JAMA Health Forum*. Published June 06, 2025. doi:10.1001/jamahealthforum.2025.1430

Data
Data available: No

Additional Information
Explanation for why data not available: The JAMDAS is a proprietary database owned by M3, Inc.; therefore, it cannot be shared. Individuals who are interested in using the JAMDAS data should contact Yusuke Tsugawa (ytsugawa@mednet.ucla.edu).
